# Supplementary material for: Tead1a Initiates Transcriptional Priming Through the TEAD1a/YAP‐Notch1‐Spi1/Cebpα Axis to Promote Neutrophil Fate
Source: Adv Sci (Weinh). 2025 Aug 28;12(41):e05441. doi: 10.1002/advs.202505441 (PMC12591127; doi:10.1002/advs.202505441)
Supplement: Supplementary file 4 — Supplementary Dataset [file ADVS-12-e05441-s004.zip › 3-7.zip!/3-7/02-7.cluster/cluster19/Enrich/ego_CC.pdf]

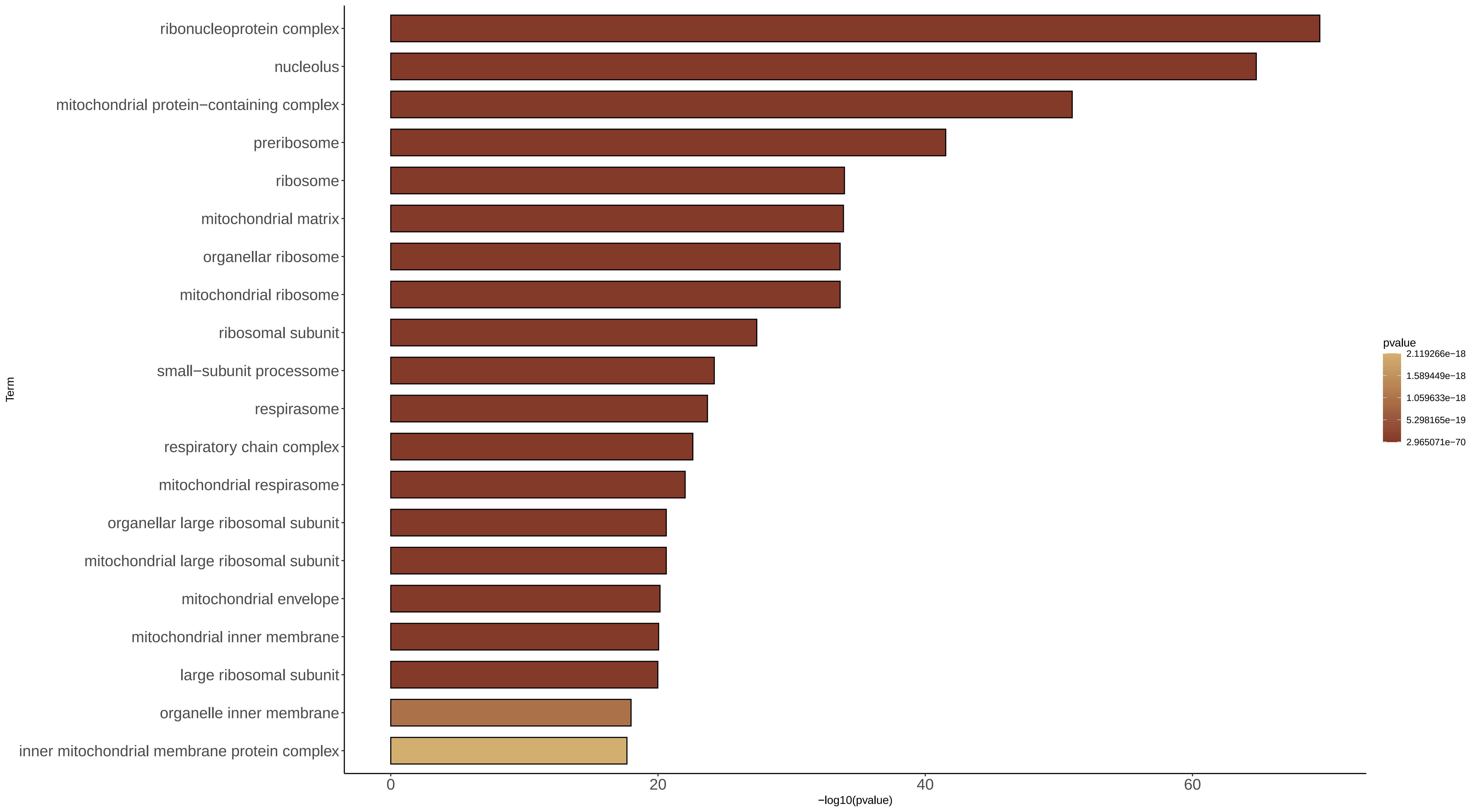
ribonucleoprotein complex
nucleolus
mitochondrial protein−containing complex
preribosome
ribosome
mitochondrial matrix
organellar ribosome
mitochondrial ribosome
ribosomal subunit
small−subunit processome
respirasome
respiratory chain complex
mitochondrial respirasome
organellar large ribosomal subunit
mitochondrial large ribosomal subunit
mitochondrial envelope
mitochondrial inner membrane
large ribosomal subunit
organelle inner membrane
inner mitochondrial membrane protein complex
Term
0
20
40
60
−log10(pvalue)
pvalue
2.119266e−18
1.589449e−18
1.059633e−18
5.298165e−19
2.965071e−70
